# Retraction: Moderating role of compassion in the link between fear of Coronavirus disease and mental health among undergraduate students

**DOI:** 10.3389/fpsyt.2023.1285013

**Published:** 2023-09-04

**Authors:** Frontiers Editorial Office

The Publisher retracts the cited article.

Following publication, concerns were raised regarding the contributions of the authors of the article. Our investigation, conducted in accordance with Frontiers policies, confirmed a serious breach of our authorship policies and of publication ethics; the article is therefore retracted.

This retraction was approved by the Chief Editors of Frontiers in Psychiatry and the Chief Executive Editor of Frontiers. The authors have not responded to correspondence regarding this retraction.

